# Enhancing the ROS generation ability of a rhodamine-decorated iridium(iii) complex by ligand regulation for endoplasmic reticulum-targeted photodynamic therapy†

**DOI:** 10.1039/d0sc04751a

**Published:** 2020-10-12

**Authors:** Lihua Zhou, Fangfang Wei, Jingjing Xiang, Hongfeng Li, Chunbin Li, Pengfei Zhang, Chuangjun Liu, Ping Gong, Lintao Cai, Keith Man-Chung Wong

**Affiliations:** Guangdong Key Laboratory of Nanomedicine, Shenzhen Engineering Laboratory of Nanomedicine and Nanoformulations, CAS Key Lab for Health Informatics, Shenzhen Institutes of Advanced Technology, Chinese Academy of Sciences Shenzhen 518055 P. R. China ping.gong@siat.ac.cn pf.zhang@siat.ac.cn; Department of Chemistry, Southern University of Science and Technology 1088 Xueyuan Blvd. Shenzhen 518055 P. R. China keithwongmc@sustech.edu.cn; College of Chemistry and Pharmaceutical Engineering, Huanghuai University 463000 Zhumadian China liuchuangjun@huanghuai.edu.cn; School of Applied Biology, Shenzhen Institute of Technology No. 1 Jiangjunmao Shenzhen 518116 P. R. China

## Abstract

The endoplasmic reticulum (ER) is a very important organelle responsible for crucial biosynthetic, sensing, and signalling functions in eukaryotic cells. In this work, we established a strategy of ligand regulation to enhance the singlet oxygen generation capacity and subcellular organelle localization ability of a rhodamine-decorated iridium(iii) complex by variation of the cyclometallating ligand. The resulting metal complex showed outstanding reactive oxygen species generation efficiency (1.6-fold higher than that of rose bengal in CH_3_CN) and highly specific ER localization ability, which demonstrated the promise of the metal-based photo-theranostic agent by simultaneously tuning the photochemical/physical and biological properties. Additionally, low dark cytotoxicity, high photostability and selective tumour cell uptake were featured by this complex to demonstrate it as a promising candidate in photodynamic therapy (PDT) applications. *In vivo* near infrared fluorescence (NIRF) imaging and tumour PDT were investigated and showed preferential accumulation at the tumour site and remarkable tumour growth suppression, respectively.

## Abstract 2

A design strategy for boosting the ROS generation of rhodamine-decorated cyclometallated iridium(iii) complexes by ligand regulation for endoplasmic reticulum-targeted precise photodynamic therapy.

## Graphical abstract

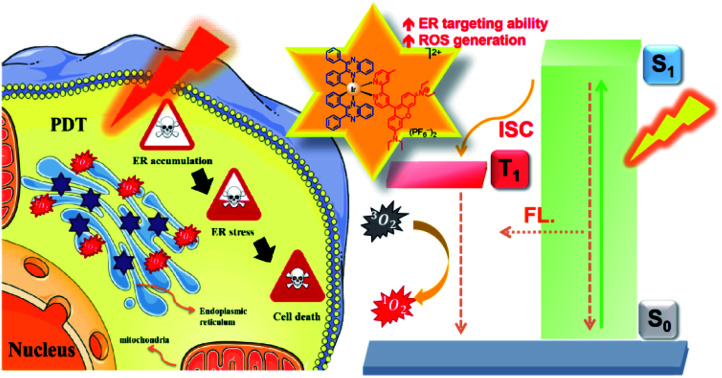

## Introduction

Significant advancements in cancer research have been achieved since the last decade, while precise cancer therapy remains a great challenge.^1–3^ Because of their minimally invasive nature, high spatiotemporal precision, controllability, and localized treatment, a great number of photo-theranostic systems have been exploited and used in cancer treatment.^4–6^ Photodynamic therapy (PDT) is a clinically non-invasive cancer phototherapy approach that uses photosensitizers (PSs) and light to convert molecular oxygen into highly reactive and damaging reactive oxygen species (ROS).^7,8^ An ideal PS for precise cancer phototherapy is expected to possess several inherent characteristics, including a large molar extinction coefficient (*ε*), highly efficient ROS generation ability, outstanding photostability and good biocompatibility.^7,9,10^ Porphyrin-based small molecules, such as haematoporphyrin derivatives, have been widely used as FDA-approved photosensitizers for clinical photodynamic therapy.^11^ It has been reported PS with subcellular organelle targeting capability show better performance for cancer phototherapy.^12^ However, conventional PSs usually suffer from some defects, such as insufficient ROS production and inefficient organelle-targeting ability. In view of these drawbacks, there has been ongoing interest in the development of new PSs with better ROS generation and organelle localization capability in order to enhance PDT performance.^7,9,12^

One of the strategic designs for efficient PSs can be rationalized by improvement in the triplet excited state population for the generation of ROS through the introduction of heavy atoms as a result of the heavy-atom effect, such as Br, I or transition metals into the chromophores^13–15^ In recent years, luminescent transition metal complexes with suitable excited states have been known to be promising photo-theranostic candidates owing to their superior photochemical and photophysical properties,^16–20^ such as (i) an efficient intersystem crossing (ISC) from the singlet (S_1_) to triplet (T_1_) excited state; (ii) a long-lived triplet excited state for allowing the interaction with the surrounding reactant before going back to its ground state; (iii) the versatility of a ligand or metal centre for the variation of photochemical and photophysical properties; and (iv) the existence of various excited states that can be accessed with visible light. More importantly, many transition metal complexes, particularly Ru(ii) and Ir(iii) complexes, exhibit intense and long-lived red to near-infrared phosphorescence with less interference and auto-fluorescence in the application of *in situ* monitoring of PS distribution in tumour cells. Such excited state properties offer novel theranostic platforms for image-guided PDT and aid in the mechanistic study of PDT in order to optimize treatment efficacy.^21–24^

Organelle targeting has recently emerged as a promising strategy in developing effective and precise cancer therapeutics.^25^ In this respect, some researchers have developed various strategies for targeting different organelles of the nucleus,^26,27^ mitochondria,^28,29^ lysosomes,^30,31^ and the endoplasmic reticulum (ER).^16,32,33^ Among these subcellular organelles for targeted therapy, related studies of the ER have rarely been explored due to their complexity in cell signalling. As the largest cellular organelle, the ER is responsible for crucial biosynthetic, sensing, and signalling functions in eukaryotic cells. Particularly, the ER has been suggested to be involved in the synthesis, folding, and post-translational modifications of proteins destined for the secretory pathway, which accounts for approximately 30% of the total proteome.^34^ ROS produced by PDT process in ER area would destroy ER protein-folding capacity which resulted in excessive or aberrant ER stress, and ultimately activated apoptotic signalling pathways causing cell death.^35^ Therefore, selective disruption of ER function in cancer cells is a promising strategy for anticancer therapy.^36,37^

**Scheme 1:**
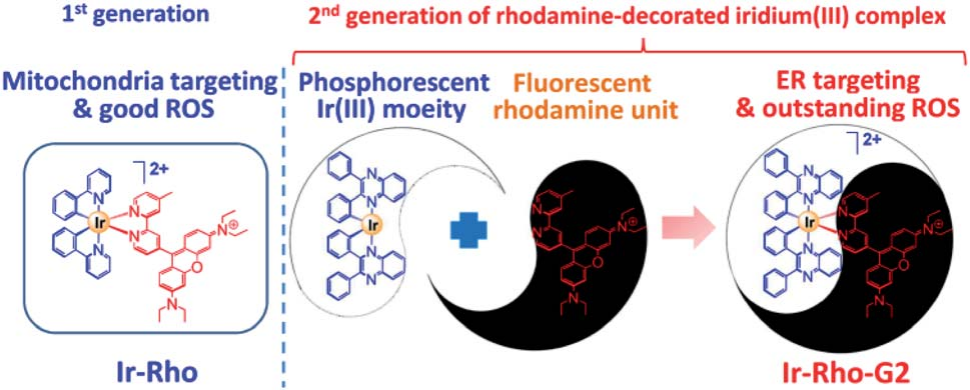
Rhodamine-decorated iridium(iii) complex, **Ir-Rho-G2**, with endoplasmic reticulum targeting and enhanced singlet oxygen generation showing high efficiency-induced apoptosis of cancer cells for efficient PDT.

In our previous study, we developed a versatile strategy to generate a series of rhodamine-decorated transition metal complexes of rhenium(i), iridium(iii), rhodium(iii) and platinum(ii) as mitochondria-targeting photosensitizers for efficient PDT.^38^ Among these rhodamine–metal complexes, one with an iridium(iii) metal centre, ([Ir(ppy)_2_(**bpy-Rho**)](PF_6_)_2_ (ppy = 2-phenylpyridine), named **Ir-Rho**) (Scheme 1) demonstrated the best performance as a photosensitizer for such organelle-localized PDT. However, **Ir-Rho** is still in its infancy, and further improvement of ROS generation efficiency is anticipated. Based on our previous findings that the population of the long-lived triplet excited state of rhodamine through intersystem crossing (ISC) is crucial in the formation of ROS for photocytotoxicity ability, both the rhodamine-appended ligand (**bpy-Rho**) and the third-row transition metal iridium are considered the key components for such PDT behaviour. On the basis of previous photophysical properties of the related luminescent cyclometallated iridium(iii) complex,^39^ the energy level, lifetime and nature of the lowest-lying excited state could be readily tuned by variation of the cyclometallating ligand. At the same time, the subcellular localization of the complex may also be modulated through a change in hydrophobicity by such ligand modification. Cho *et al.* has reported a dual functional molecular dyad, from the combination of cyclometallated Ir(iii) complex and rhodamine B, which showed lysosomal staining and photodynamic therapy.^40^ Our group has explored a bichromophoric rhodamine-tethered iridium(iii) sensory system with the cyclometallating ligand 2,3-diphenylquinoxaline (dpqx). Such hybrid system exhibited interesting controllable energy transfer process from rhodamine to iridium(iii) luminophore.^41^ Recently, a series of highly luminescent rhodium(iii) complexes with the same dpqx ligand were reported by us. Impressive photoluminescence quantum yield of 65% in solid state and respectable external quantum yield of 12.2% were achieved^42^ The choice of this lower-lying dqpx ligand is anticipated to modulate the photoluminescence properties, in particular of tuning the energy level and lifetime of transition metal triplet excited state. In view of these aspects, a programme based on modification of the cyclometallating ligand 2,3-diphenylquinoxaline (dpqx) with a lower-lying excited state aiming to further improve the PDT performance was launched to afford the second generation of **Ir-Rho**, [Ir(dpqx)_2_(**bpy-Rho**)](PF_6_)_2_ named herein as **Ir-Rho-G2** (Scheme 1). In particular, the singlet oxygen generation capacity and ER targeting ability of **Ir-Rho-G2** were significantly enhanced, and efficient PDT performance was found as a result of the disruption of ER function leading to cancer cell death upon light irradiation.

## Results and discussion

The strategy for improving the singlet oxygen generation ability and organelle targeting behaviour of the rhodamine-containing cyclometallated iridium(iii) system was achieved by simply replacing the cyclometallating ligand from 2-phenylpyridine (ppy) to 2,3-diphenylquinoxaline (dpqx) in **Ir-Rho-G2**. According to the previously reported procedure of **Ir-Rho**,^38^**Ir-Rho-G2** was synthesized from reaction of the corresponding iridium(iii) dimeric starting material, [Ir(dpqx)Cl]_2_ and the rhodamine-tethered bipyridine chelating ligand, **bpy-Rho**. The characterization of **Ir-Rho-G2** was confirmed by ^1^H and ^13^C NMR and high-resolution mass spectrometry (Fig. S1 and S2†), as well as satisfactory of elemental analysis results.

**Fig. 1:**
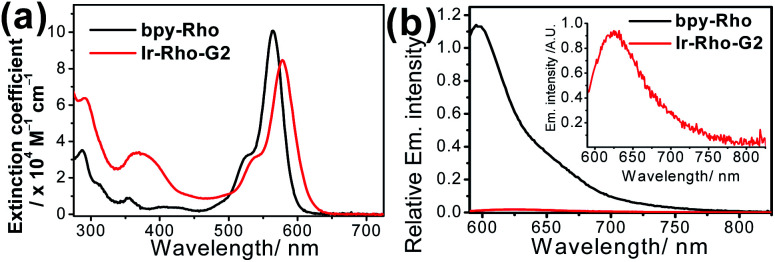
(a) UV/vis absorption and (b) emission spectra of **bpy-Rho** and **Ir-Rho-G2** in CH_3_CN (ex. at 550 nm with abs. = 0.6).

The lifetime of the excited state involving the iridium(iii) metal centre, such as metal-to-ligand charge transfer (MLCT) or metal-perturbed intraligand (IL) charge transfer, was reported to enhance luminescence quantum yield (*Φ*_lum_).^43^ In order to test our strategy, an extended π-conjugated dpqx cyclometallating ligand with a lower-lying intraligand (IL) state was employed in this study. Additionally, the lower excitation and luminescence energies upon variation of this ligand are advantageous for biological applications and compatibility. The photophysical behaviours of **Ir-Rho-G2** have been investigated in order to corroborate our idea of strategic design. The photophysical data of **bpy-Rho**, **Ir-Rho** and **Ir-Rho-G2** are summarized in Table S1.† Their electronic absorption and emission spectra (Fig. 1) in acetonitrile showed a low-energy absorption band at 578 nm and very weak fluorescence at 635 nm, respectively. With reference to our previously reported rhodamine-containing transition metal system,^38^ such low-energy absorption and emission are assigned as the corresponding characteristic rhodamine-based absorption and fluorescence. Compared to those of the free ligand of **bpy-Rho**, it is noteworthy that the absorption and fluorescence of **Ir-Rho-G2** were red-shifted, which strongly suggests direct coupling of the metal centre and π-conjugation core of rhodamine unit upon incorporation of iridium(iii) metal scaffold. In contrast, some rhodamine-containing transition metal systems,^40,41,44,45^ were reported to show negligible red-shift of the rhodamine absorption because of the poor interaction between rhodamine and metal centres.

**Scheme 2:**
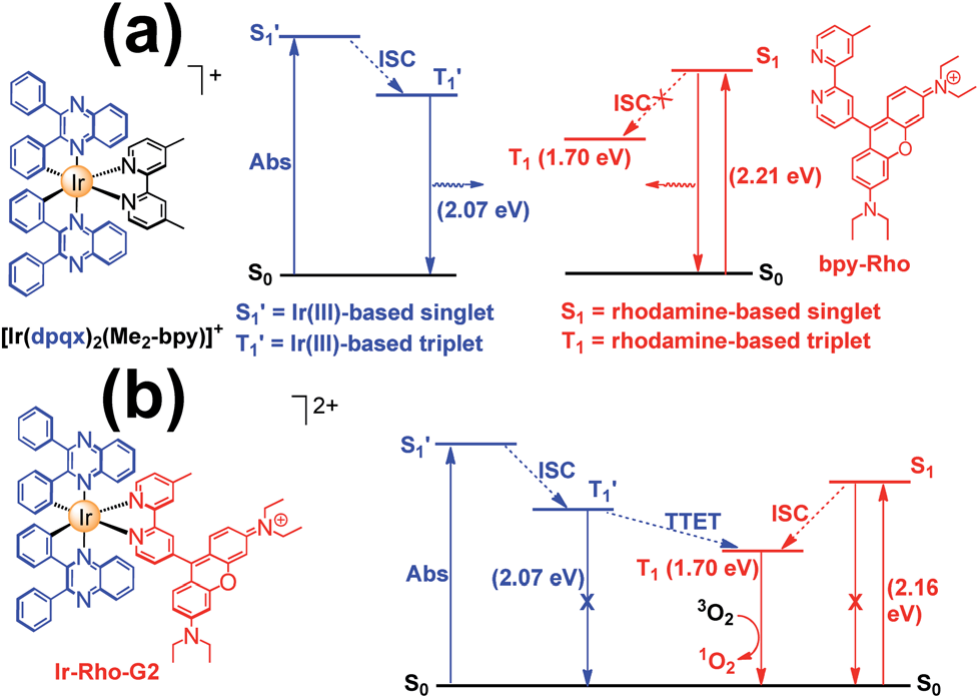
Qualitative energy diagram of (a) [Ir(dpqx)_2_(Me_2_-bpy)](PF_6_) and **bpy-Rho**; and (b) **Ir-Rho-G2** illustrating the population of the rhodamine-based triplet excited state (T_1_). The energy levels of S_1_ and 
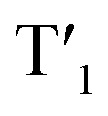
 were estimated by the on-sets of their emission energies.

The energy levels of cylcometallated iridium(iii)-based 
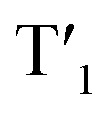
 and rhodamine S_1_ could be estimated from the emissions of their corresponding components, *i.e.*, phosphorescence from rhodamine-free [Ir(dpqx)_2_(Me_2_-bpy)](PF_6_) and fluorescence from rhodamine-tethered chelate **bpy-Rho**, respectively (Scheme 2a). Upon connection of these two components in **Ir-Rho-G2**, no observable iridium(iii)-based phosphorescence and very weak rhodamine fluorescence were detected, regardless of the excitation at iridium(iii)-based absorption or rhodamine absorption band. It is suggested that the triplet excited state (T_1_) of the rhodamine unit would be populated as the lowest excited state in rhodamine-containing transition metal complexes (Scheme 2b). This population was mainly derived from two sensitization processes: (I) triplet–triplet energy transfer (TTET) from the iridium(iii)-based triplet state 
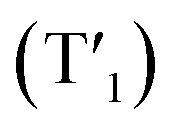
 and (II) intersystem crossing (ISC) from the rhodamine singlet state (S_1_). The occurrence of TTET is rationalized from the lower energy level of T_1_ (approximately 1.70 eV)^38^ than that of iridium(iii)-based 
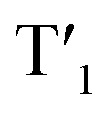
 (1.89 eV). On the other hand, the luminescence quantum yield (*Φ*_lum_) from rhodamine fluorescence supports the efficient population of T_1_ from S_1_. Zhao and co-workers have reported a number of examples showing population of triplet state from various organic fluorophores, including coumarin, BODIPY, naphthalimide, naphthalene diimide and perylenebisimide, through ISC in the corresponding transition metal incorporated systems.^13^ The criteria for efficient ISC is either by direct metallation into the organic fluorophore, or by connection of transition metal and the organic fluorophore *via* π-conjugation. **Ir-Rho-G2** was suggested to fulfill the second criterion in order to undergo efficient ISC from singlet state to triplet state of rhodamine moiety. The singlet-to-triplet energy transfer from rhodamine singlet state to ^3^MLCT excited state is unflavoured in **Ir-Rho** because the energy level of ^3^MLCT excited state of [Ir(ppy)_2_(bipy)]^+^ is higher than that of rhodamine singlet state.^38^ However, this spin-forbidden energy transfer in **Ir-Rho-G2** cannot completely be ruled out, in view of the lower-lying ^3^MLCT excited state with dpqx ligand. In contrast, no such ISC was observed in other rhodamine-containing transition metal systems, in that there are no direct interactions between the metal centre and the rhodamine.^40,41,44,45^ As a result, strong residual rhodamine fluorescence and negligible absorption shift upon incorporation of transition metal centres were featured in these systems. On the basis of this, the judicious choice of linker is crucial for the interaction between metal centre and rhodamine moiety in order to facilitate the ISC of rhodamine by heavy atom effect.

**Fig. 2:**
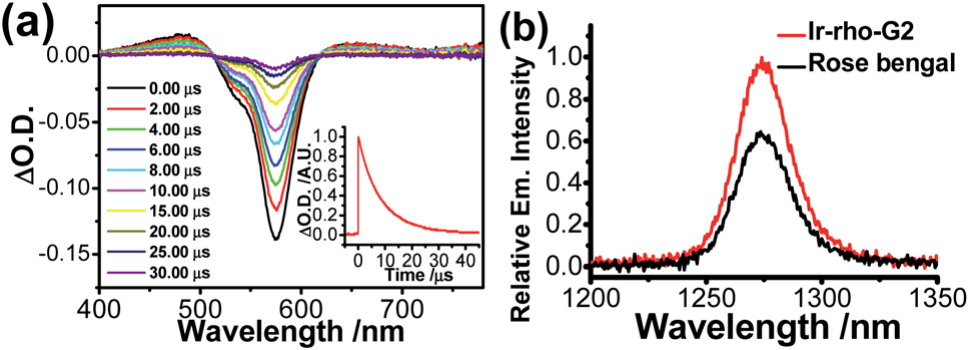
(a) Nanosecond time-resolved transient difference absorption spectra of **Ir-Rho-G2** in deaerated CH_3_CN; *λ*_ex_ = 355 nm. The inset shows the decay trace of the signal at 575 nm. (b) Singlet oxygen emission spectra of **Ir-Rho-G2** and rose bengal in CH_3_CN with an excitation wavelength of 514.5 nm.

Although rhodamine-based T_1_ is in a dark state, the presence of this state could be probed by using nanosecond transient difference (ns-TA) spectroscopy. The characteristic bleaching signal of rhodamine absorption at 575 nm, attributed to the rhodamine T_1_ state, was observed in the time-resolved TA spectra of **Ir-Rho-G2** (Fig. 2a). The lifetime of this T_1_ state in **Ir-Rho-G2** was determined to be 9.73 μs (inset of Fig. 2a), which is approximately 10 times longer than the T_1_ lifetime of **Ir-Rho** (0.82 μs). Because of the prolonged triplet excited state, the ability of ^1^O_2_ generation was investigated by monitoring the emission of ^1^O_2_ at 1270 nm upon excitation at 514.5 nm. Stronger ^1^O_2_ emission intensity and ^1^O_2_ generated quantum yield (*Φ*_Δ_) were observed for **Ir-Rho-G2** (72.6%) in CH_3_CN (Fig. 2b) compared to those of **Ir-Rho** (43.0%) as well as the commonly used and commercially available standard photosensitizer rose bengal (45.0%).^46^ In principle, a higher *Φ*_Δ_ will be predicted for compounds with a lower luminescence quantum yield (*Φ*_lum_). The observation of a higher *Φ*_Δ_ and a lower *Φ*_lum_ (0.07%) for **Ir-Rho-G2** relative to those of **Ir-Rho** (*Φ*_Δ_, 43.0%; *Φ*_lum_, 1.4%) demonstrated our strategy design through ligand modification for the enhancement of ROS generation ability. It is noteworthy that noticeable changes were not observed for the absorbance of **Ir-Rho-G2**, while the absorbance of rose bengal decreased dramatically (Fig. S3†). This result strongly indicates that **Ir-Rho-G2** is more photostable with higher resistance to photobleaching compared with rose bengal.

**Fig. 3:**
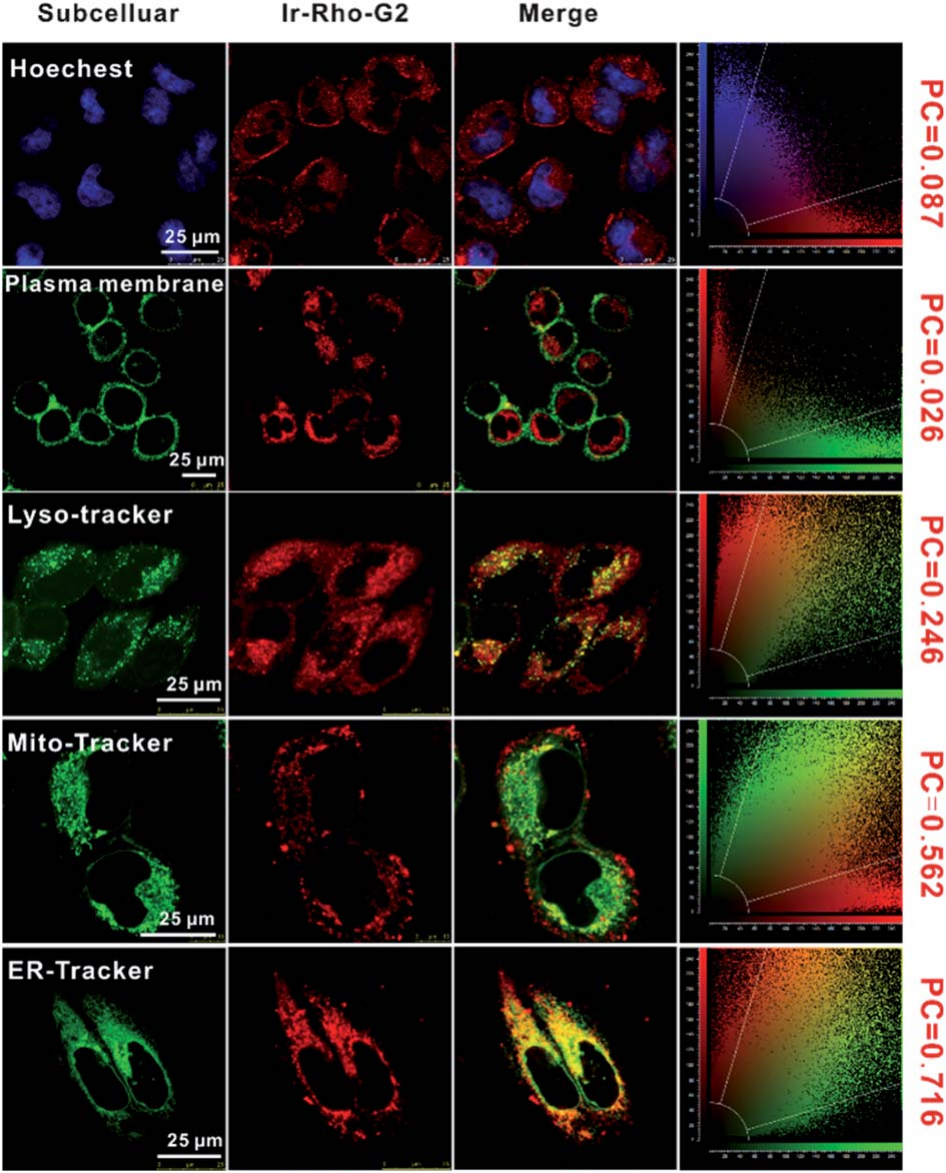
Confocal laser scanning microscopy images of MCF-7 cells treated with **Ir-Rho-G2** (5 μM, *λ*_ex_ = 561 nm, *λ*_em_ = 600–700 nm) for 30 min and then stained with the Hoechst (nucleus stain, *λ*_ex_ = 405 nm, *λ*_em_ = 420–490 nm), CellMask™ Green (plasma membrane stain, *λ*_ex_ = 488 nm, *λ*_em_ = 510–560 nm), LysoTracker™ Green DND-26 (lysosome stain, *λ*_ex_ = 488 nm, *λ*_em_ = 510–560 nm), MitoTracker Green (mitochondria stain, *λ*_ex_ = 488 nm, *λ*_em_ = 510–560 nm), ER-Tracker™ Green (endoplasmic reticulum stain, *λ*_ex_ = 488 nm, *λ*_em_ = 510–560 nm), respectively.

The intracellular localization of **bpy-Rho**, **Ir-Rho** and **Ir-Rho-G2** were investigated by confocal fluorescence microscopy. Since **Ir-Rho** and **Ir-Rho-G2**, which are comprised of the same rhodamine-tethered ligand and iridium(iii) metal centre, are structurally similar and with the same charge of +2, specific mitochondrial localization was also anticipated for **Ir-Rho-G2**, as was observed for **Ir-Rho**.^41^ Upon co-staining with a mito-specific probe, essentially different microscopic images of them were observed (Fig. S4†). The Pearson's Coefficient (PC) value of **Ir-Rho-G2** (0.562) with Mitotracker is much lower than those of **Ir-Rho** (0.934) and **bpy-Rho** (0.809). On the contrary, confocal microscopy imaging, upon co-staining with various organelle-specific probes, indicated that **Ir-Rho-G2** is mainly and specifically localized in the endoplasmic reticulum (ER) with PC value of 0.716 (Fig. 3). The corresponding overlap of fluorescence signals between the compounds and other trackers suggests that the introduction of the 2,3-diphenylquinoxaline ligand in **Ir-Rho-G2** resulted in the dominantly specific intracellular localization to the ER, whereas **bpy-Rho** and **Ir-ppy** were revealed to be relatively more localized to the mitochondria (Fig. S4†). Moreover, when **Ir-Rho-G2** was co-incubated with an ER-tracker in MCF-7 cells, competitive uptake between them was observed (Movie S1†), probably due to the increased hydrophobicity in **Ir-Rho-G2** (Table S1†) upon the change of the cyclometallated ligand.

**Fig. 4:**
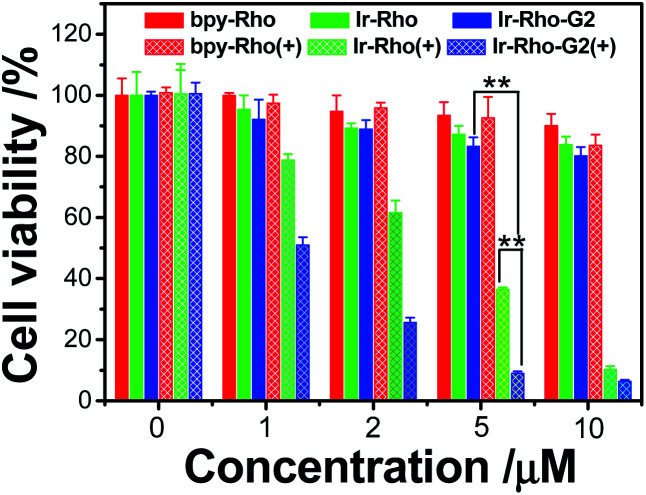
Survival of MCF-7 cells after treatment with **bpy-Rho**, **Ir-Rho** and **Ir-Rho-G2** at different concentrations. No irradiation (solid bar) and after irradiation with an 11 W lamp (grid bar). MCF-7 cells were seeded at 8 × 10^3^ to 1 × 10^4^ cells per well in 96 well plates then incubated for 24 h in 5% CO_2_ at 37 °C. The solutions of the complexes (10 mM in DMSO) were diluted to appropriate concentrations with the complete medium and added to the cells to give final complexes concentrations (1, 2, 5, 10 μM). After incubation for 2 h, the well plate was irradiated with 11 W lamp for 30 min. After the irradiation, the cells were again incubated for 4 h, after which the cytotoxicity was determined by CCK8 assay and expressed as a percent of the controls (cells exposed to light in the absence of the complexes).

To explore the PDT effects of **Ir-Rho-G2** upon ligand modification, *in vitro* PDT studies of **Ir-Rho-G2** together with **bpy-Rho** and **Ir-Rho** were carried out on MCF-7 cancer cells. Intracellular ROS production upon light irradiation was first tested and monitored by the fluorescence intensity measurements using the DCF assay. As shown in Fig. S5,† intracellular ROS were significantly generated by evaluation of the DCF fluorescence. In line with the photophysical results in solution, **Ir-Rho-G2** was found to have the highest ROS generation ability under the same conditions. The PDT therapeutic effects of **bpy-Rho**, **Ir-Rho** and **Ir-Rho-G2** in MCF-7 cells by CCK8 assay were accordingly tested. Negligible or only small changes in cell viability were observed without light irradiation, suggesting their low cytotoxicity in the dark. Upon irradiation with an 11 W lamp, the cell viability gradually decreased with increasing concentrations of **Ir-Rho-G2** (Fig. 4). Significant cell death was observed for **Ir-Rho-G2** at concentrations as low as 2 μM. The cytotoxic effects of **Ir-Rho-G2** were approximately three times better than that of our previously reported **Ir-Rho**. One of the main reasons suggested is due to its higher ^1^O_2_ production as measured above.

**Fig. 5:**
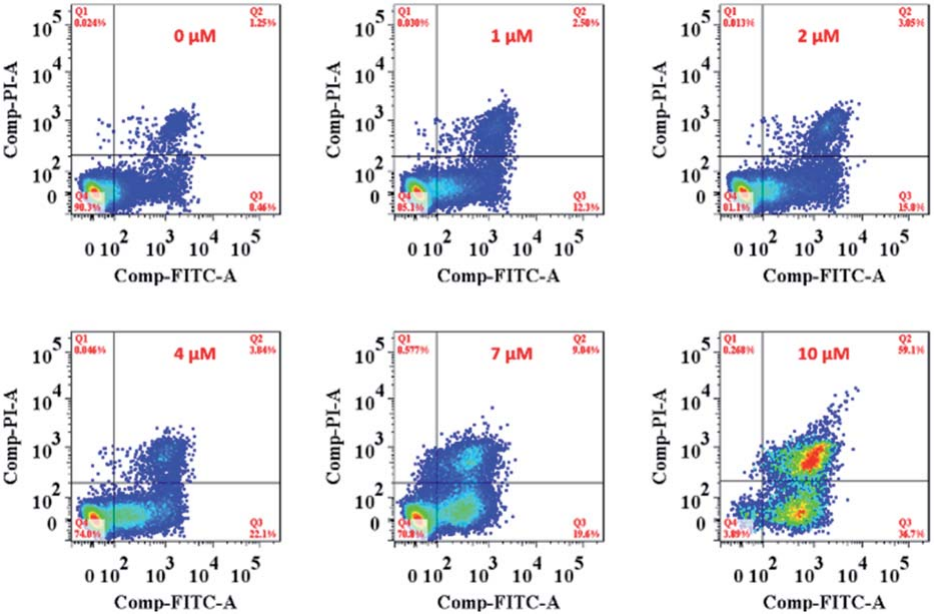
Flow cytometry analysis of MCF-7 cell apoptosis after treatment with **Ir-Rho-G2** at different concentrations. MCF-7 cells were seeded at 8 × 10^3^ to 1 ×10^4^ cells per well in 96 well plates then incubated for 24 h in 5% CO_2_ at 37 °C. The solutions of the complexes (10 mM in DMSO) were diluted to appropriate concentrations with the complete medium and added to the cells to give final complexes concentrations (1, 2, 5, 10 μM). After incubation for 2 h, the well plate was irradiated with 11 W lamp for 30 min. After the irradiation, the cells were again incubated for 4 h, after which the cell apoptosis was determined by standard flow cytometry analysis approach.

On the other hand, the ER is responsible for crucial biosynthetic, sensing, and signalling functions in eukaryotic cells. The mechanism of cell death in our study is probably the result of disturbing the protein-folding capacity of the ER, ultimately activating apoptotic signalling pathways and cell death. The flow cytometry analysis cell apoptosis results further supported that **Ir-Rho-G2** was found to activate apoptotic signalling pathways and cell death (Fig. 5 and Movie S2†).

**Fig. 6:**
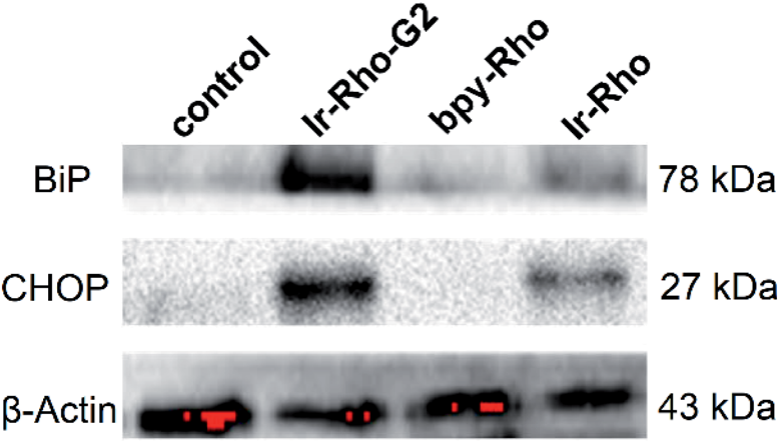
Western blot analysis of the ER stress markers BiP and CHOP after treating MCF-7 cells with control (PBS), **bpy-Rho**, **Ir-Rho** and **Ir-Rho-G2** (5 μM) under irradiation with an 11 W lamp for 30 min.

Since the ER is the major site of protein synthesis and processing, accumulation of **Ir-Rho-G2** and the generated ROS may induce ER dysfunction. To prove this hypothesis, time-dependent western blotting was employed to examine the expression of markers of ER stress. In this process, the C/EBP homologous protein (CHOP, a downstream sensor of severe ER stress) and the binding immunoglobulin protein (BiP, an Hsp70 family chaperone localized in the ER lumen) expression levels will be upregulated to a high-level during ER stress-induced apoptosis. To validate the ER stress caused by **Ir-Rho-G2**, western blot analysis was conducted to trace the proteins extracted from **bpy-Rho**, **Ir-Rho** and **Ir-Rho-G2** in pre-treated MCF-7 cells. Treating MCF-7 cells with **Ir-Rho-G2** after 11 W lamp irradiation was confirmed to increase the levels of the BiP and CHOP (Fig. 6). In contrast, the expression levels of BiP and CHOP in the cells treated with **bpy-Rho** and **Ir-Rho** remained low. The results suggested that **Ir-Rho-G2** can cause the upregulation of CHOP and BiP after PDT, which highlights its promising potential as an ER-localized PDT photosensitizer candidate.

A variety of cell lines, including normal cell lines (MCF-10A/bEnd3/L02/293T) and tumour cell lines (MCF-7/A549/HepG2/4T1), were used for the intracellular uptake study of **Ir-Rho-G2** by flow cytometry. The results showed that the **Ir-Rho-G2** fluorescence intensity in tumour cell lines was approximately 1 to 3 times stronger than that of other normal cell lines (Fig. S6†), which demonstrated the highest uptake efficiency and confirmed the stronger self-recognition affinity of **Ir-Rho-G2** to tumour cells. Such results may be caused by a stronger ability to metabolize in tumour cells than in normal cells. Previous studies have reported that some organic small molecules with a positive charge have characteristics of preferential tumour-specific accumulation. This is mainly attributed to the transportation of these dyes into tumour cells by organic-anion transporting polypeptides (OATPs) that would be overexpressed on the surface of many tumour cells. The lipotropic cation property of **Ir-Rho-G2** probably resulted in a higher uptake efficiency in tumour cells than in normal cells.

**Fig. 7:**
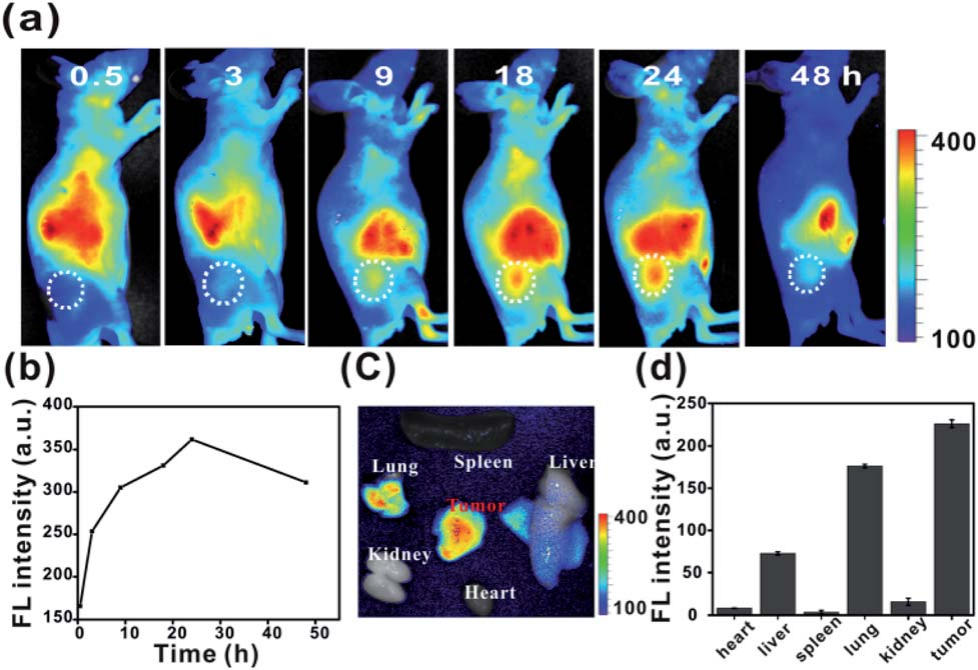
(a and b) *In vivo* NIRF imaging of **Ir-Rho-G2** (200 μM, 150 μL) in MCF-7 tumour-bearing nude mice from 0 to 48 h. (c) *Ex vivo* NIR images and (d) fluorescence intensity of the dissected organs and tumours 24 h post-injection.

To investigate the *in vivo* tumour-targeting ability of **Ir-Rho-G2**, *in vivo* near infrared fluorescence (NIRF) images are shown in Fig. 7. The fluorescence signal was primarily observed in the whole body at 0.5 h post-injection. The signal at the tumour site could be distinguished from the surrounding normal tissue at 9 h post-injection and increased gradually to reach a maximum at 24 h post-injection (Fig. 7a and b). *Ex vivo* fluorescence biodistribution revealed that **Ir-Rho-G2** preferentially accumulated at the tumour site compared to the normal heart, liver, spleen, lung and kidney tissues (Fig. 7c and d).

**Fig. 8:**
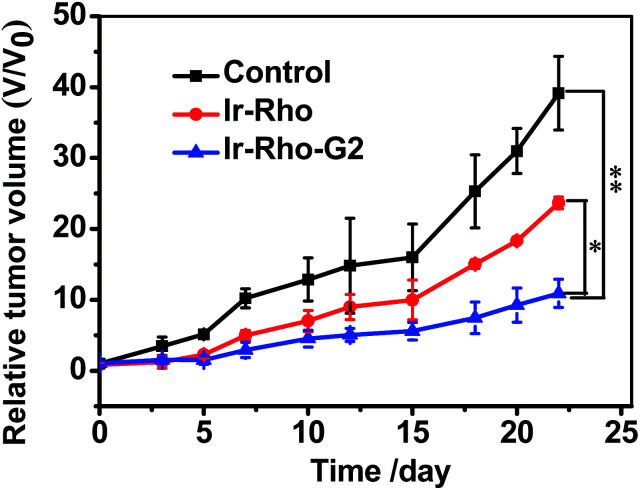
*In vivo* PDT therapy using **Ir-Rho** and **Ir-Rho-G2**. When the tumors had reached an average volume of 100 mm^3^, mice (*n* = 5) were intravenously injected with saline (200 μL), **Ir-Rho** and **Ir-Rho-G2** (200 μL, 10 μM) separately. Xenon lamp light source system was used to irradiate the tumors for 30 min for two times at 24 and 48 hours after injection. The growth of the tumor was measured with a caliper every 2 or days during the period of treatment (25 days) and the volume was calculated (volume = length × width^2^ × 0.5). The percentage of surviving mice was determined by monitoring tumor growth-related events. The mice were assumed to be death when tumor size over 1000 mm^3^.

On the basis of the excellent tumour cell selectively and NIRF imaging *in vivo* and the highly efficient PDT of **Ir-Rho-G2***in vitro*, we further applied **Ir-Rho-G2** for tumour PDT *in vivo*. **Ir-Rho-G2** and **Ir-Rho** were intravenously injected into tumour-bearing mice when the tumour size grew to 50–100 mm^3^. Since the accumulation of **Ir-Rho-G2** and **Ir-Rho** in tumours reached a maximum at 15 h after injection, as previously revealed by NIRF imaging, mice were irradiated at the tumour site at that time with a xenon lamp light source for 30 min. The tumour volume was measured every 2 or 3 days to monitor the rate of tumour growth. Compared to **Ir-Rho**, the mice treated with **Ir-Rho-G2** showed remarkable tumour growth suppression and valuable tumour regression (Fig. 8). And no tumor recurrence was observed with 100% survival rate during the observation period (Fig. S7a†). Hence, it was noteworthy that **Ir-Rho-G2** exhibited robust PDT therapeutic efficacy. There was no obvious variation in mice weight in all treated groups, suggesting that the experimental treatments were well tolerated (Fig. S7b†). To further investigate the *in vivo* the **Ir-Rho-G2** toxicity to other organs after treatment, H&E stained images of sliced major organs collected from different groups were obtained. No noticeable abnormalities were observed in the major organs, including the liver, spleen, kidney, heart, and lung, from the PDT-treated mice, suggesting negligible toxic side effects of **Ir-Rho-G2** after PDT *in vivo* (Fig. S8†). The *in vivo* bio-safety of the **Ir-Rho** and **Ir-Rho-G2** was investigated by liver/kidney function index, including alanine aminotransferase (ALT), aspartate aminotransferase (AST), blood urea nitrogen (BUN), and creatinine (CRE). As shown in Fig. S9,† the concentration of ALT/AST/BUN/CRE showed no obvious change compared with control group, demonstrating the remarkable biosafety of **Ir-Rho-G2***in vivo*.

## Experimental section

### Materials and reagents

All the solvents for the synthesis were of analytical grade.

### General procedure for the synthesis of Ir-dqpx-Rho

A mixture of {Ir(dqpx)_2_Cl}_2_ (0.08 mmol) and **bpy-Rho** (0.16 mmol) in 20 mL of methanol/dichloromethane (1 : 1 v/v) was refluxed under an inert atmosphere of nitrogen in the dark for 12 h. The solution was then cooled to room temperature, and KPF_6_ (0.16 mmol) was added to the solution. The mixture stirred for 30 min at room temperature and then evaporated to dryness. The residue was purified by column chromatography (silica gel; CH_2_Cl_2_/CH_3_OH). Yield 147 mg (60%). ^1^H NMR (400 MHz, CD_3_CN) *δ* (ppm) 9.04 (d, *J* = 5.6 Hz, 1H), 8.70 (d, *J* = 5.7 Hz, 1H), 8.12 (s, 1H), 8.04 (dd, *J* = 7.2, 4.7 Hz, 2H), 7.96 (s, 1H), 7.94 (m, 2H), 7.89 (d, *J* = 7.0 Hz, 3H), 7.74 (m, 5H), 7.70–7.64 (m, 4H), 7.53 (d, *J* = 8.7 Hz, 2H), 7.41–7.36 (m, 1H), 7.26 (d, *J* = 7.2 Hz, 1H), 7.22 (d, *J* = 8.9 Hz, 1H), 7.17 (d, *J* = 8.2 Hz, 1H), 7.10 (d, *J* = 9.6 Hz, 1H), 7.05–7.01 (m, 1H), 6.86 (d, *J* = 2.2 Hz, 1H), 6.82–6.76 (m, 3H), 6.76–6.64 (m, 5H), 6.60 (d, *J* = 6.9 Hz, 1H), 3.67 (q, *J* = 14.2, 7.0 Hz, 4H), 3.57 (q, *J* = 7.4 Hz, 4H), 2.41 (s, 3H), 1.28 (t, *J* = 7.1 Hz, 6H), 1.20 (t, *J* = 7.0 Hz, 6H). ^13^C NMR (126 MHz, CD_3_CN) *δ* (ppm) 163.70, 163.62, 157.76, 157.70, 156.68, 155.89, 155.76, 154.34, 154.30, 154.22, 153.19, 152.93, 152.63, 150.39, 149.38, 148.15, 144.74, 144.55, 144.17, 140.90, 140.84, 140.79, 140.50, 139.79, 139.73, 135.52, 135.21, 131.50, 131.04, 130.87, 130.73, 130.47, 130.44, 130.40, 130.11, 130.07, 129.97, 129.88, 129.26, 129.18, 129.12, 125.50, 124.49, 124.35, 124.30, 122.45, 122.41, 114.81, 114.41, 112.29, 112.21, 96.40, 45.85, 20.36, 11.85. HRMS (ESI). Calcd for C_74_H_63_ON_6_Ir ([M − 2PF_6_]^2+^), *m*/2 623.2294; found, *m*/2 623.2298.

### Physical measurements and instrumentation

The UV-vis absorption spectra were taken on a Cary 60 UV-vis spectrophotometer. Steady-state emission spectra at room temperature were recorded on an Edinburgh Instruments FLS980 fluorescence spectrometer. Quartz cuvettes (path length = 1 cm) were used in all spectrophotometric and fluorometric measurements. NMR spectra were recorded on a Bruker AVANCE 400 (^1^H NMR for 400 MHz and ^13^C NMR for 100 MHz) Fourier transform NMR spectrometer with chemical shifts reported relative to tetramethylsilane, (CH_3_)_4_Si. High-resolution MS spectra were obtained on an Orbitrap Fusion Tribrid mass spectrometer. Elemental analyses of the newly synthesized complexes were performed on an Elementar Vario EL Cube elemental analyser at Sun Yat-sen University. The nanosecond time-resolved transient-difference absorption spectra were detected by using an Edinburgh LP920 instrument (Edinburgh Instruments, U.K.). All solutions for transient absorption studies were degassed with a high-vacuum line in a two-compartment cell consisting of a 10 mL Pyrex bulb and a 1 cm path length quartz cuvette and sealed from the atmosphere by a Bibby Rotaflo HP6 Teflon stopper. The solutions were rigorously degassed with at least four successive freeze–pump–thaw cycles. Luminescence quantum yields were measured with a Hamamatsu C11347 absolute photoluminescence quantum yield measurement system.

Singlet oxygen emission was detected by using an FLS-980 spectrofluorometer. All of the compounds were dissolved in CH_3_CN. The absorbance at 514.5 nm, as the excitation wavelength, was adjusted to be approximately 0.35 for **Ir-Rho-G2** and rose bengal. For the measurements, an 850 nm long-pass filter was inserted between the sample and the detector to avoid high-order diffraction from the visible emission. The singlet oxygen quantum yield was determined by comparing the ^1^O_2_ emission intensity of rose bengal upon excitation at 514.5 nm (*Φ*_Δ_ = 45% in CH_3_CN).^46^

A photostability test was performed by using a 532 nm laser with 336 mW cm^−2^ output power as the irradiation source. The compounds were dissolved in CH_3_CN, and their absorbance was checked after 0, 1, 3, 5, 7, and 10 min. Their absorbance at 532 nm was normalized at time = 0 s.

### Cell culture

MCF-7 human breast cancer cells, A549 human lung cancer cells, HepG2 hepatocellular carcinoma cells, 293T human kidney cells and 4T1 mouse breast cancer cells were cultured in DMEM (Gibco) supplemented with 10% fetal bovine serum (FBS) (Gibco), 1% penicillin and 1% streptomycin. Cells were incubated at 37 °C in a humidified incubator with 5% CO_2_. MCF-10A human breast cells, L02 human liver cells and NIH-3T3 mouse embryonic fibroblasts were maintained in RPMI-1640 medium supplemented with 10% fetal bovine serum (FBS) and 1% penicillin and streptomycin. Cells were incubated at 37 °C in a humidified incubator with 5% CO_2_.

### Subcellular localization

MCF-7 cells (5000 cells) were cultured in eight-well chambered coverglasses (Lab-Tek, Nunc, USA) for 24 h. Later, the cells were incubated with **Rho-bpy**, **Ir-Rho**, **Ir-Rho-G2** (final concentration = 5 μM). After incubation for 30 min, the cells were treated with MitoTracker for 10 min to specifically stain the mitochondria (1 : 5000 dilution in PBS). After washing three times with PBS, confocal microscopy imaging was performed.

### Selective uptake

Tumour cells (MCF-7/A 549/HepG2/4T1) and normal cells (MCF-10A/293T/L02/NIH-3T3) (1 × 10^4^ cells) were cultured in six-well plates for 12 h. Then, the medium of the cells was changed to medium containing 5 μM **Ir-Rho-G2** complexes. Cells were washed three times with PBS after 2 h of incubation. Then, the cells were harvested, and the fluorescence intensity of complexes was recorded by flow cytometry.

### Cytotoxicity

MCF-7 cells were seeded at 8 × 10^3^ to 1 ×10^4^ cells per well in 96-well plates and then incubated for 24 h in 5% CO_2_ at 37 °C. Solutions of the complexes (10 mM in DMSO) were diluted to the appropriate concentrations with complete medium and added to the cells to give final complex concentrations of 1, 2, 5, and 10 μM. After incubation for 2 h, the well plate was irradiated with an 11 W lamp for 30 min. After irradiation, the cells were again incubated for 4 h, after which the cytotoxicity was determined by CCK8 assay and expressed as a percent of the control (cells exposed to light in the absence of the complexes).

### Cells apoptosis

The MCF-7 cells with different concentrations of **Ir-Rho-G2** incubated 30 min and then were irradiated with 11 W lamp 30 min, MCF-7 cells were collected by careful trypsinization and low-speed centrifugation and washed twice with PBS. The cells were resuspended in 100 μL of binding buffer and stained with 3 μL of annexin V-FITC and 5 μL of PI for 15 min at room temperature in the dark. After being stained, the cells were collected by low-speed centrifugation, washed twice with PBS, and diluted with 600 μL of binding buffer for flow cytometry analysis (Becton Dickinson, San Jose, CA, USA).

### Animals and tumour model

Animals received care in accordance with the Guidance Suggestions for the Care and Use of Laboratory Animals. The procedures were approved by the Animal Care and Use Committee (Shenzhen Institutes of Advanced Technology, Chinese Academy of Sciences). Six- to eight-week-old female BALB/c mice or nude mice (Vital River Laboratory Animal Technology Co. Ltd., China) were subcutaneously injected with MCF-7 cells (1 × 10^6^) in the flank region.

### *In vivo* imaging

A Maestro GNIR Flex imaging system and analysis with software from CRi were used to image tumour-bearing animals over time following i.v. treatment with **Ir-Rho-G2** (200 μM, 150 μL). At each time point, the mouse was imaged at 600 nm excitation and above 650 nm emission. At 24 h post injection, tumours and major organs were excised for *ex vivo* NIRF imaging to determine the tissue distribution of Rho-Ir-dpqx. All main organs were imaged at 600 nm excitation and above 650 nm emission. The average fluorescence intensity of organs was from a region of interest (ROI) placed over the area of the organs on the image.

### *In vivo* antitumor efficacy studies

When the tumors had reached an average volume of 100 mm^3^, mice (*n* = 5) were intravenously injected with saline (200 μL), **Ir-Rho** and **Ir-Rho-G2** (200 μL, 10 μM) separately. Xenon lamp light source system was used to irradiate the tumors for 30 min for two times at 24 and 48 hours after injection. The growth of the tumor was measured with a caliper every 2 or days during the period of treatment (25 days) and the volume was calculated (volume = length × width^2^ × 0.5). The percentage of surviving mice was determined by monitoring tumor growth-related events. The mice were assumed to be death when tumor size over 1000 mm^3^.

### Statistical analysis

All the results are reported as mean ± SD. The differences among groups were determined using one-way ANOVA analysis and student's *t*-test; *P < 0.05, **P < 0.01.

## Conclusions

In summary, we reported a design strategy to improve the PDT performance of a rhodamine-decorated iridium(iii) complex, **Ir-Rho-G2**, by variation of the cyclometallating ligand with the lowest-lying excited state for the elongation of lifetime. **Ir-Rho-G2** was found to exhibit outstanding reactive oxygen species generation efficiency in both solution and intracellular media. Moreover, the subcellular localization of the complex may also be altered to possess ER targeting ability, which is suggested to be correlated to cancer cell death through apoptosis upon light irradiation. Additionally, low dark cytotoxicity, high photostability and selective tumour cell uptake were observed for this complex. *In vivo* NIRF imaging and tumour PDT were investigated and showed preferential accumulation at the tumour site and remarkable tumour growth suppression. This work opens up a new avenue for the exploitation of promising phototheranostic agents with highly efficient ROS generation and organelle localization capability for precise cancer therapy by the strategy of ligand regulation.

## Conflicts of interest

There are no conflicts of interest to declare.

## Supplementary Material

SC-011-D0SC04751A-s001

SC-011-D0SC04751A-s002

SC-011-D0SC04751A-s003
