# Supplementary material for: Enhancing the ROS generation ability of a rhodamine-decorated iridium(iii) complex by ligand regulation for endoplasmic reticulum-targeted photodynamic therapy
Source: Chem Sci. 2020 Oct 12;11(44):12212–20. doi: 10.1039/d0sc04751a (PMC8162876; doi:10.1039/d0sc04751a)
Supplement: SC-011-D0SC04751A-s001 [file SC-011-D0SC04751A-s001.pdf]

Electronic Supplementary Material (ESI) for Chemical Science.
This journal is © The Royal Society of Chemistry 2020

Electronic Supplementary Material (ESI) for Chemical Science.

This journal is © The Royal Society of Chemistry 2018

Electronic Supplementary Material (ESI) for Chemical Science.

This journal is © The Royal Society of Chemistry 2018

SUPPORTING INFORMATION

Enhancing the ROS Generation Ability of a Rhodamine-Decorated Iridium(III) Complex by Ligand-Regulation for Endoplasmic Reticulum-Targeted Photodynamic Therapy

Lihua Zhou,[‡a, d] Fangfang Wei,[‡b] Jingjing Xiang,[a] Hongfeng Li,[a] Chunbin Li,[a] Pengfei Zhang,*[a] Chuangjun Liu,*[b,c] Ping Gong,*[a] Lintao Cai[a] and Keith Man-Chung Wong*[b]

[a] Guangdong Key Laboratory of Nanomedicine, Shenzhen, engineering Laboratory of nanomedicine and nanoformulations, CAS Key Lab for Health Informatics, Shenzhen Institutes of Advanced Technology, Chinese Academy of Sciences, Shenzhen 518055, P. R. China. ping.gong@siat.ac.cn, pf.zhang@siat.ac.cn

[b] Department of Chemistry, Southern University of Science and Technology, 1088 Xueyuan Blvd., Shenzhen 518055, P.R. China. keithwongmc@sustc.edu.cn

[c] College of Chemistry and Pharmaceutical Engineering, Huanghuai University, 463000 Zhumadian, China. liuchuangjun@huanghuai.edu.cn

[d] School of Applied Biology, Shenzhen Institute of Technology, No.1 Jiangjunmao, Shenzhen 518116, P.R. China.

Table S1 Photophysical Data of Ir-Rho-G2, together with bpy-Rho, Ir-Rho.

	λ_{abs}, nm (MeCN)	ε, M^{-1} cm^{-1}	λ_{em}, nm (MeCN)	Φ_{em}, %	τ, μs	Φ_{Δ}, %	C logP
Ir-Rho-G2	578	68100 [a]	635	0.7 [b]	9.73 [c]	72.6 [d]	11.178 [e]
bpy-Rho [f]	564	98600	598	29.0	n.d.	0.3	3.108 [e]
Ir-Rho [f]	575	87500	629	1.4	0.82	43.0	6.778 [e]

[a] Molar extinction coefficient at the absorption maxima. [b] Emission quantum yield. [c] Triplet-state lifetime measured by transient absorption in anaerobic CH_3CN. [d] Singlet oxygen quantum yield, relative to rose bengal (Φ_{Δ} = 0.45 in MeCN), from the emission peak with excitation wavelength at 514.5 nm. [e] C logP calculated by Chemdraw. [f] From ref. 38.

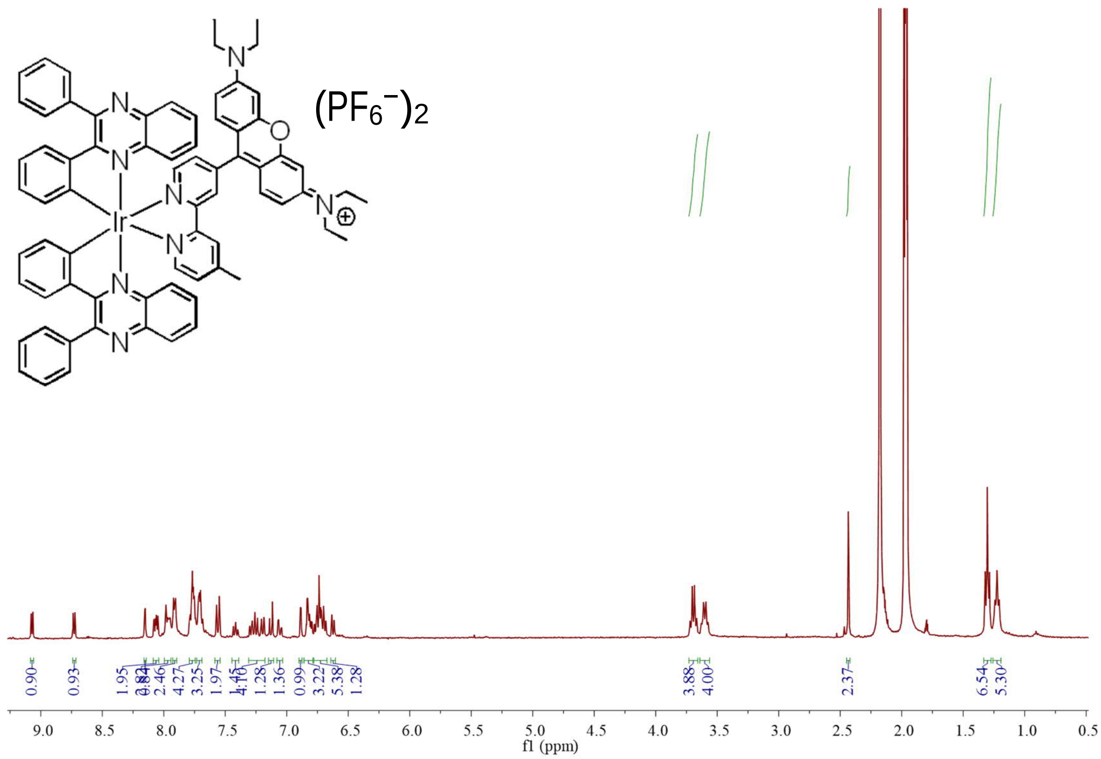

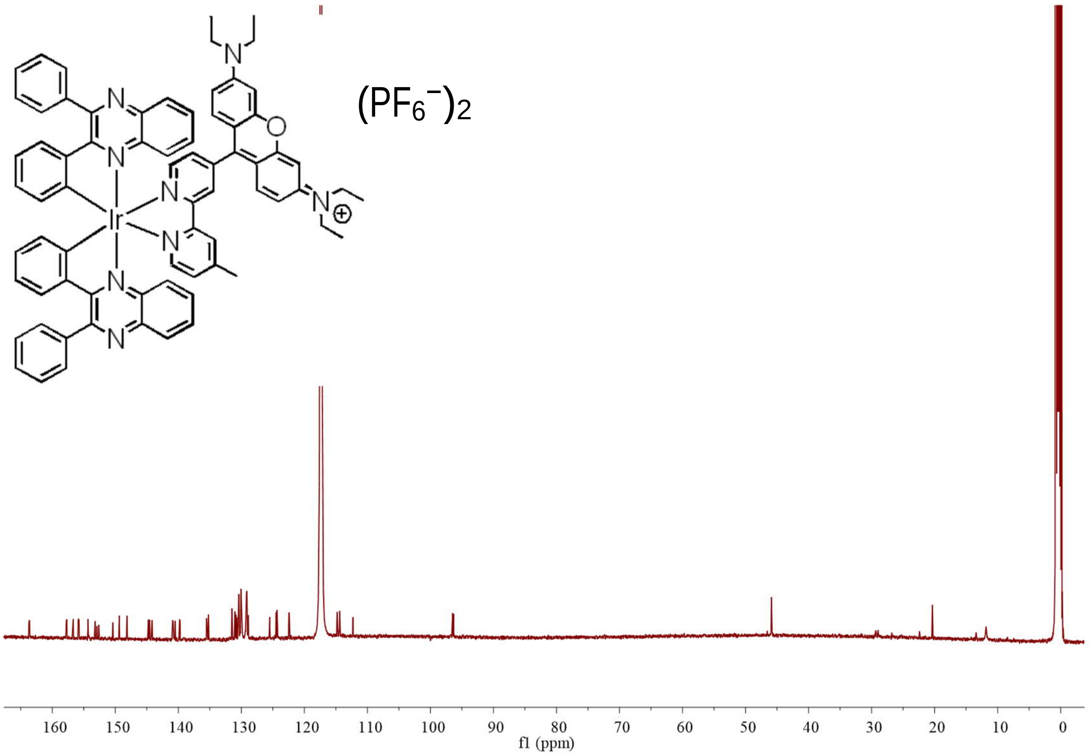

Figure S1. ^{1}H (top) and ^{13}C (bottom) NMR spectra of Ir-Rho-G2 in CD_3CN at 298K.

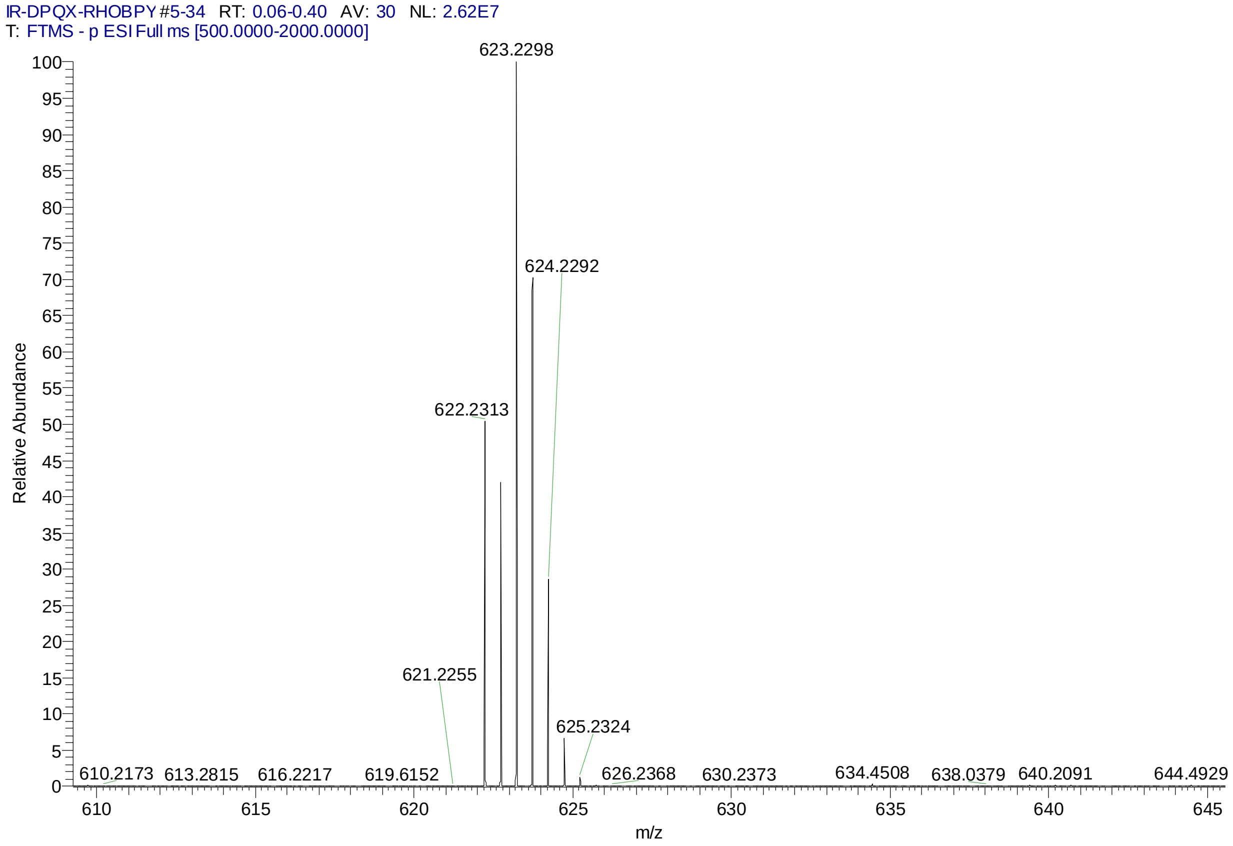

Figure S2. high-resolution mass spectroscopy of Ir-Rho-G2.

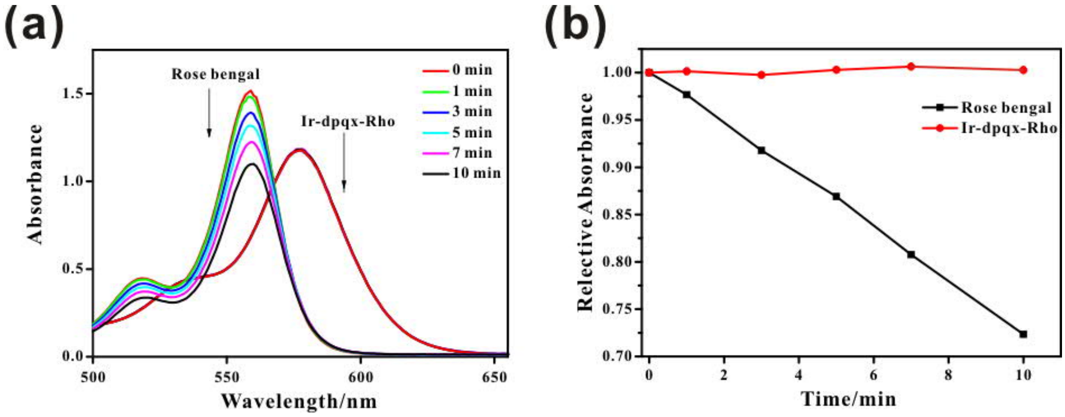

Figure S3. Photostability study of Ir-Rho-G2 and Rose Bengal in their UV-Vis absorption spectra (a) and the corresponding relative absorbance (b) using 532 nm laser with 336 mW/cm^2. The absorbance monitored for Ir-Rho-G2 and Rose Bengal are 562 nm and 580 nm, respectively.

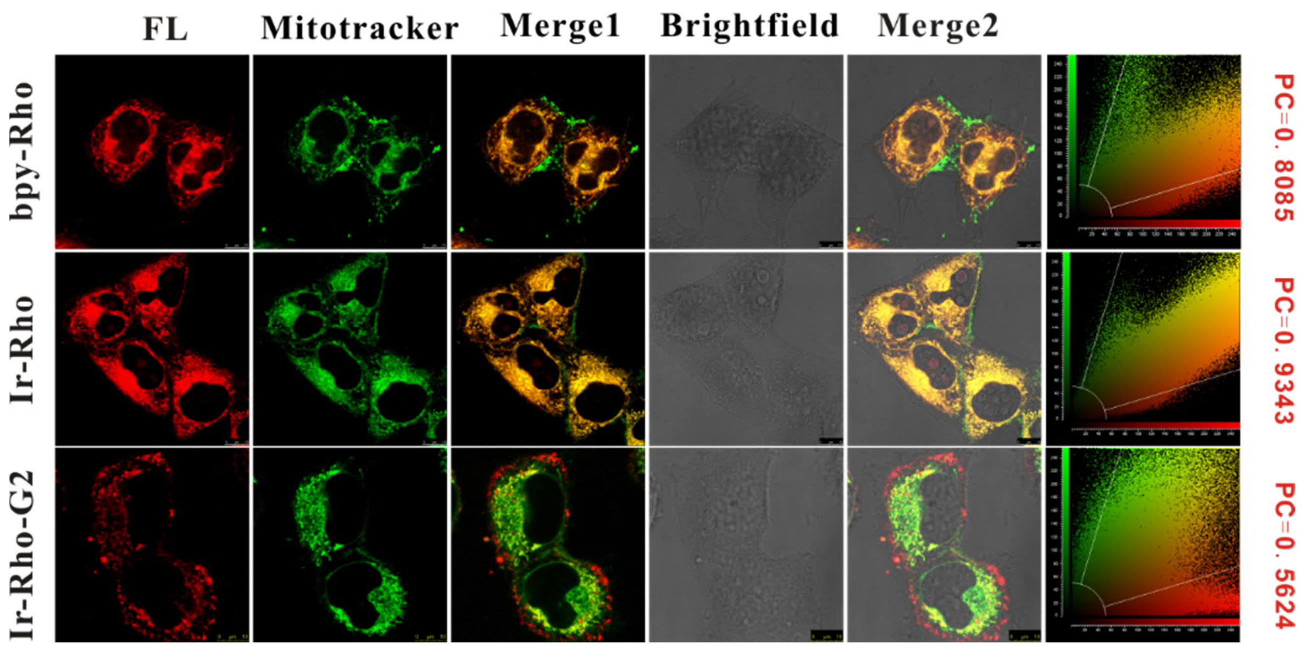

Figure S4. Subcellular colocalization in MCF-7 tumor cells after treating with bpy-Rho, Ir-Rho and Ir-Rho-G2 and MitoTracker Green.

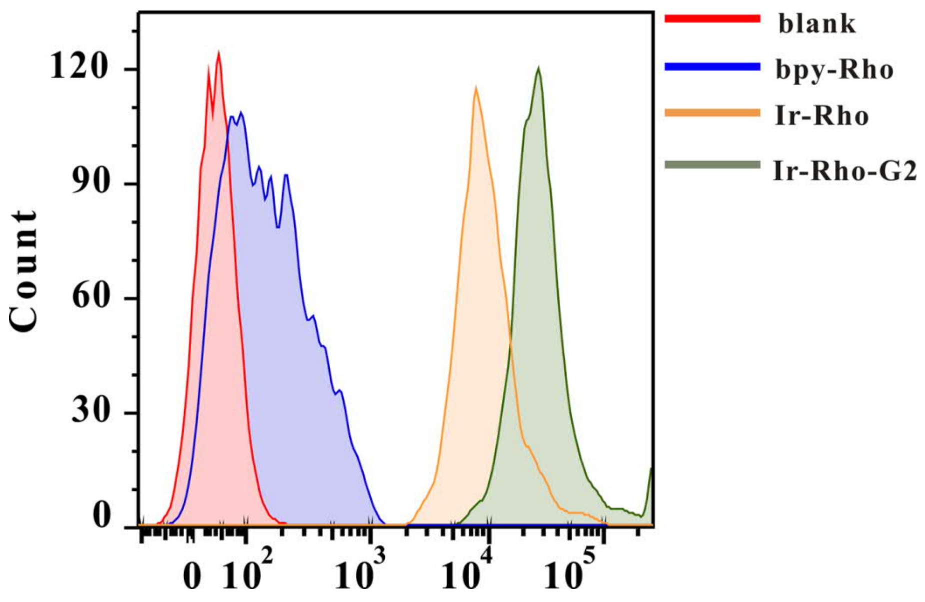

Figure S5. DCFH-DA assay for the evaluation of intracellular ROS production of bpy-Rho, Ir-Rho and Ir-Rho-G2 (5 μM) in DMEM; incubation with MCF-7 cells in the dark for 30 min followed by 30 min irradiation flow cytometry analysis (n = 10 000 cells) with mean fluorescence intensity per cell.

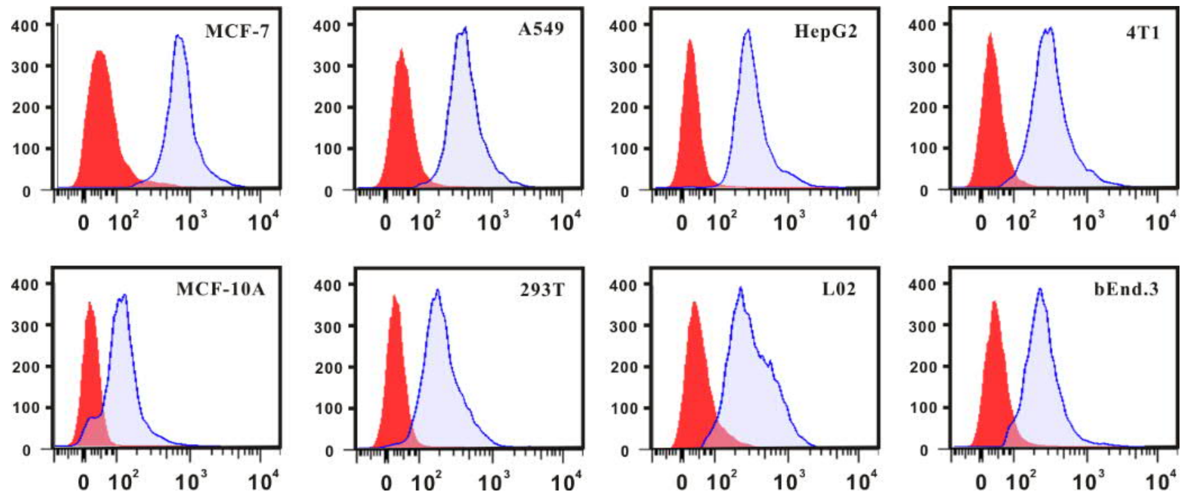

Figure S6. Flow cytometry analysis (n = 10000 cells) of Ir-Rho-G2 in normal cells (MCF-10A, 293T, L02, bEnd3,) and tumor cells (MCF-7, A549, HepG2, 4T1,) with normalized fluorescence intensity.

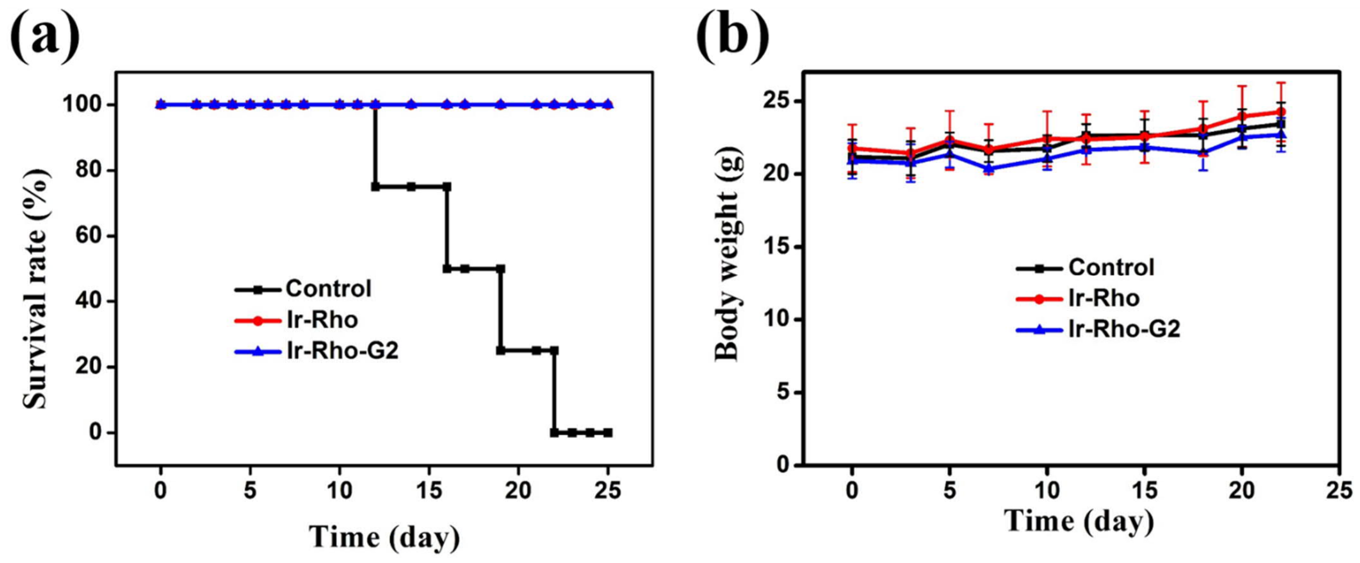

Figure S7. In vivo PDT therapy using Ir-Rho and Ir-Rho-G2. (a) average weights and (b) survival rate profiles of the mice bearing MCF-7 tumors in different groups for 22 days observation period after treatment.

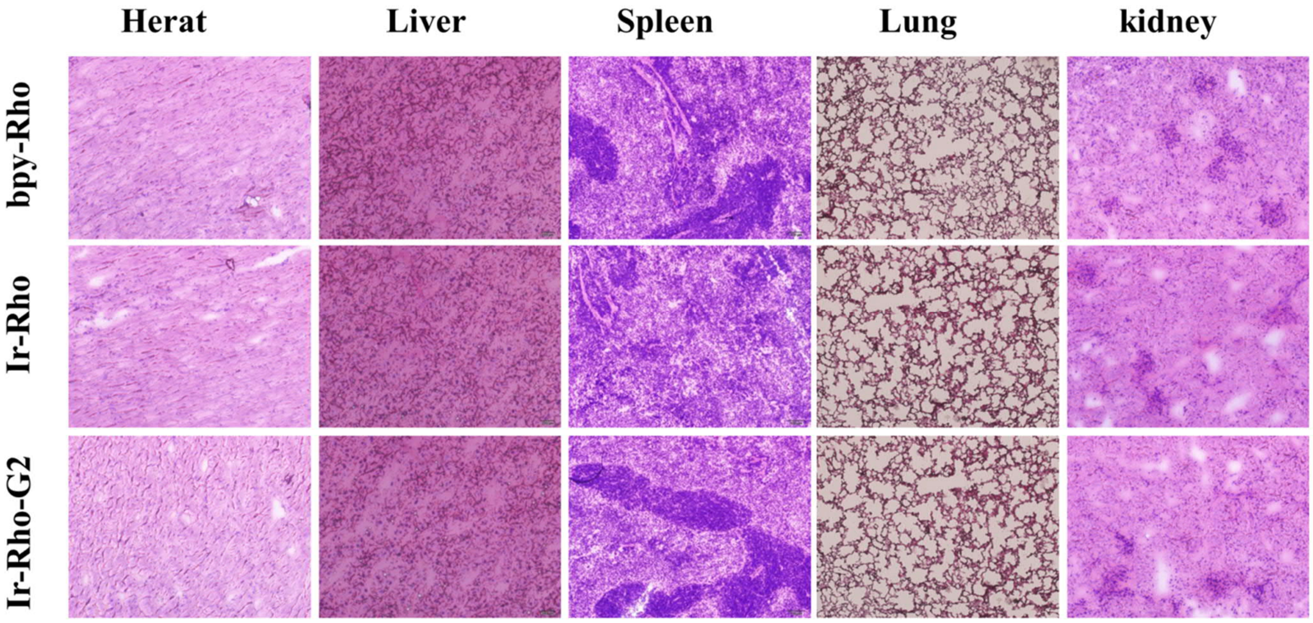

Figure S8. H&E stained images of sliced major organs collected from different groups.

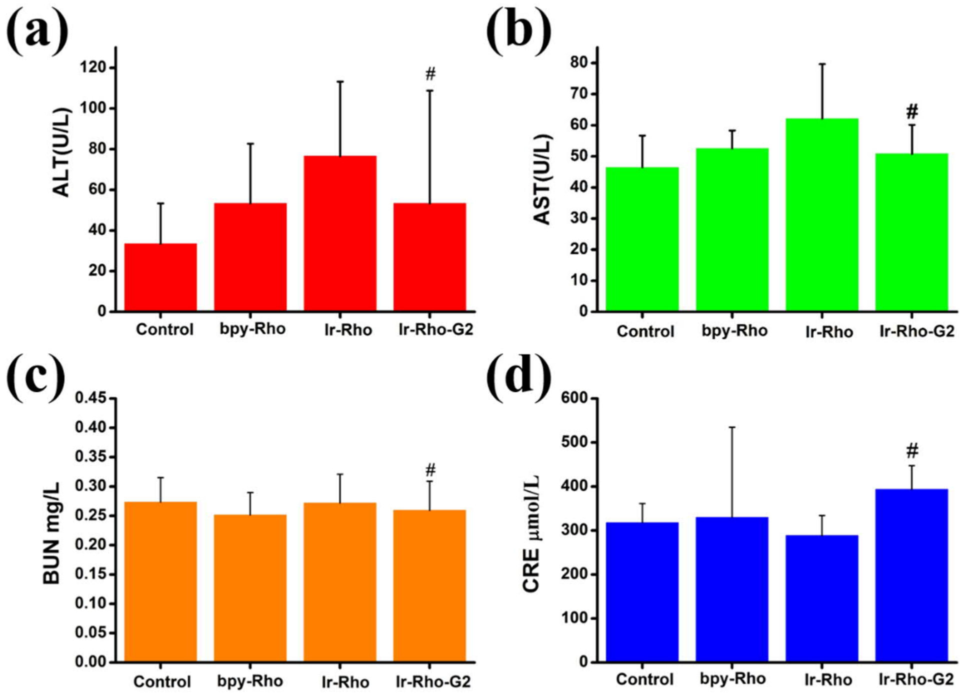

Figure S9. Nude mouse serum levels of ALT, AST, BUN, and CRE after intravenous injecting Ir-Rho and Ir-Rho-G2. Error bars are standard error of the mean ($^{\#}P<0.05$) as compared to control.
